# Nicotinic Receptors in the Brainstem Ascending Arousal System in SIDS With Analysis of Pre-natal Exposures to Maternal Smoking and Alcohol in High-Risk Populations of the Safe Passage Study

**DOI:** 10.3389/fneur.2021.636668

**Published:** 2021-03-10

**Authors:** Arunnjah Vivekanandarajah, Morgan E. Nelson, Hannah C. Kinney, Amy J. Elliott, Rebecca D. Folkerth, Hoa Tran, Jacob Cotton, Perri Jacobs, Megan Minter, Kristin McMillan, Jhodie R. Duncan, Kevin G. Broadbelt, Kathryn Schissler, Hein J. Odendaal, Jyoti Angal, Lucy Brink, Elsie H. Burger, Jean A. Coldrey, Johan Dempers, Theonia K. Boyd, William P. Fifer, Elaine Geldenhuys, Coen Groenewald, Ingrid A. Holm, Michael M. Myers, Bradley Randall, Pawel Schubert, Mary Ann Sens, Colleen A. Wright, Drucilla J. Roberts, Laura Nelsen, Shabbir Wadee, Dan Zaharie, Robin L. Haynes, PASS Network

**Affiliations:** ^1^Department of Pathology, Harvard School of Medicine, Boston Children's Hospital, Boston, MA, United States; ^2^Avera Research Institute, Sioux Falls, SD, United States; ^3^Department of Pediatrics, University of South Dakota School of Medicine, Sioux Falls, SD, United States; ^4^Department of Forensic Medicine, New York University School of Medicine, New York City, NY, United States; ^5^Department of Obstetrics and Gynecology, Faculty of Medicine and Health Science, Stellenbosch University, Cape Town, South Africa; ^6^Division of Forensic Pathology, Department of Pathology, Faculty of Health Sciences, Stellenbosch University & Western Cape Forensic Pathology Service, Tygerberg, South Africa; ^7^Department of Psychiatry and Pediatrics, Columbia University Medical Center, New York State Psychiatric Institute, New York, NY, United States; ^8^Division of Genetics and Genomics and the Manton Center for Orphan Diseases Research, Boston Children's Hospital, Boston, MA, United States; ^9^Department of Pediatrics, Harvard Medical School, Boston, MA, United States; ^10^Department of Pathology, University of South Dakota Sanford School of Medicine, Sioux Falls, SD, United States; ^11^Division of Anatomical Pathology, Department of Pathology, Faculty of Medicine and Health Science, Stellenbosch University, Cape Town, South Africa; ^12^Department of Pathology, School of Medicine and Health Sciences, University of North Dakota, Grand Forks, ND, United States; ^13^Lancet Laboratories, Johannesburg, South Africa; ^14^Department of Pathology, Massachusetts General Hospital, Boston, MA, United States; ^15^MaineGeneral Health, Augusta, ME, United States

**Keywords:** acetylcholine, serotonin, cardiorespiratory, arousal, medulla oblongata, mesopontine tegmentum

## Abstract

Pre-natal exposures to nicotine and alcohol are known risk factors for sudden infant death syndrome (SIDS), the leading cause of post-neonatal infant mortality. Here, we present data on nicotinic receptor binding, as determined by ^125^I-epibatidine receptor autoradiography, in the brainstems of infants dying of SIDS and of other known causes of death collected from the Safe Passage Study, a prospective, multicenter study with clinical sites in Cape Town, South Africa and 5 United States sites, including 2 American Indian Reservations. We examined 15 pons and medulla regions related to cardiovascular control and arousal in infants dying of SIDS (*n* = 12) and infants dying from known causes (*n* = 20, 10 pre-discharge from time of birth, 10 post-discharge). Overall, there was a developmental decrease in ^125^I-epibatidine binding with increasing postconceptional age in 5 medullary sites [raphe obscurus, gigantocellularis, paragigantocellularis, centralis, and dorsal accessory olive (*p* = 0.0002–0.03)], three of which are nuclei containing serotonin cells. Comparing SIDS with post-discharge known cause of death (post-KCOD) controls, we found significant decreased binding in SIDS in the nucleus pontis oralis (*p* = 0.02), a critical component of the cholinergic ascending arousal system of the rostral pons (post-KCOD, 12.1 ± 0.9 fmol/mg and SIDS, 9.1 ± 0.78 fmol/mg). In addition, we found an effect of maternal smoking in SIDS (*n* = 11) combined with post-KCOD controls (*n* = 8) on the raphe obscurus (*p* = 0.01), gigantocellularis (*p* = 0.02), and the paragigantocellularis (*p* = 0.002), three medullary sites found in this study to have decreased binding with age and found in previous studies to have abnormal indices of serotonin neurotransmission in SIDS infants. At these sites, ^125^I-epibatidine binding increased with increasing cigarettes per week. We found no effect of maternal drinking on ^125^I-epibatidine binding at any site measured. Taken together, these data support changes in nicotinic receptor binding related to development, cause of death, and exposure to maternal cigarette smoking. These data present new evidence in a prospective study supporting the roles of developmental factors, as well as adverse exposure on nicotinic receptors, in serotonergic nuclei of the rostral medulla—a finding that highlights the interwoven and complex relationship between acetylcholine (via nicotinic receptors) and serotonergic neurotransmission in the medulla.

## Introduction

The sudden infant death syndrome (SIDS) is a major worldwide public health problem. It is defined as the sudden death of a seemingly healthy infant under 1 year of age that remains unexplained after a thorough case investigation, including the performance of a complete autopsy, an examination of the death scene, and a review of the infant's clinical history (1). Death typically occurs during sleep or during one of the many transitions to arousal that occur in normal infant sleep (2). SIDS is the leading cause of post-neonatal infant death in the United States where the overall rate is 0.35/1,000 live births (3). The SIDS risk increases in socioeconomically disadvantaged minority populations throughout the world, e.g., African-Americans in the urban United States, American Indians in the Northern Plains, mixed ancestry groups in Cape Town in South Africa, Maoris in New Zealand, and Aboriginal and Torres Strait Islanders in Australia (3–7). Biological mechanisms in minority high-risk SIDS populations have been historically understudied because of the decreased access to modern forensic centers with pediatric research tools, lack of funds for research in health disparities, and the general mistrust of autopsy by these minority populations (8–10).

A leading hypothesis in SIDS research today is that there is an abnormality in neurotransmitter networks in the lower brainstem that regulate cardiorespiratory control and arousal (11). We and others have reported abnormalities in tissue parameters of the neurotransmitter serotonin (5-HT) in the serotonergic homeostatic network in the medulla oblongata (lower brainstem) in SIDS cases compared to controls (12–17) as well as abnormalities in cholinergic (18–25), GABAergic (26), and substance P (27) networks. These abnormalities likely impair protective reflexes to life-threatening challenges during a sleep period, leading to defective arousal to a metabolic stressor (hypoxia, hypercarbia) and sleep-related sudden death. The underlying premise is that a vulnerable infant with a biological defect in homoeostasis dies suddenly in a sleep period when they fail to respond to an exogenous stressor in a critical developmental period (the Triple Risk model) (28). While the origin or basis of the biological defect is unknown, one possibility includes altered development of neurotransmitter systems due to exposure to adverse conditions *in utero*. Among these exposures, pre-natal exposure to nicotine and alcohol are candidates based on epidemiological data showing the contribution of maternal smoking (29–31) and drinking (32) to SIDS risk. Most recently, the Safe Passage Study conducted by the Pre-natal Alcohol in SIDS and Stillbirth (PASS) Network (see below) reported an increased relative risk for SIDS of 4.86 (95% CI: 0.97–24.27) for infants with pre-natal exposure to smoking only beyond the first trimester, as compared to those unexposed or those whose mothers reported quitting early in pregnancy (33). The relative risk increased to ~12-fold (98% CI: 2.59–53.7) in infants whose mothers reported both smoking and drinking beyond the first trimester, suggesting a combined, possibly synergistic, effect on infant risk (33).

The Safe Passage Study was a large, prospective, multidisciplinary study designed, in part, to investigate the association between pre-natal alcohol and/or pre-natal smoke exposure, and SIDS and stillbirth (34). A key objective of the Safe Passage Study was to elucidate the role of pre-natal exposures in altered development of neurotransmitter systems in the human brainstem. This includes development of the cholinergic receptor system in cardiorespiratory and arousal brainstem sites involved in homeostatic regulation. Acetylcholine is a neurotransmitter that mediates its effects via 2 classes of cholinergic receptors, metabotropic muscarinic receptors, and ionotropic nicotinic acetylcholine receptors (nAChRs), localized diffusely throughout the brain and brainstem. Early in fetal development, acetylcholine, via interactions with acetylcholine receptors, serves as a growth factor, affecting neuronal proliferation, growth, survival, differentiation, and pathfinding (35). Aberrant embryonic alterations in acetylcholine signaling adversely affects its morphogenetic properties during development with lasting effects into the post-natal period (35). In the Safe Passage Study, we have focused specifically on acetylcholine signaling as mediated by nAChRs because of the agonistic properties of nicotine upon binding to this receptor subtype and because of the documented effect of nicotine on nAChRs expression (36).

Nicotinic receptors are ligand-gated cation channels that exist as pentamers of subunits around a central pore. Genes encoding a total of 16 subunits (α1–10, β1–4, δ, ε, γ) have been identified in mammals (37). They are present as either homopentamers (α7, α9) or heteropentamers throughout the central and peripheral nervous system and can be found both at pre- and post-synaptic membranes (38). Human developmental studies have shown expression of many subunits as early as 4 to 5 gestational weeks, with high expression during early to midgestation, including within the brainstem (39). During early infancy, nAChR expression decreases substantially (40), suggesting a heightened vulnerability to the effects of maternal smoking during gestation. Maternal smoking results in nicotine crossing the placental barrier, the fetal blood–brain barrier, and binding to the endogenous nAChRs in the fetal brain (41) affecting nAChR expression and function. Similarly, ethanol from maternal drinking crosses the placenta into the fetal circulation and interacts with nAChRs via an ethanol binding pocket to modulate the action of the receptor (42–46). Given that both nicotine and ethanol affect the action of nAChRs within the brain, these receptors represent a common target underlying the adverse effect of maternal smoking and drinking during pregnancy. Previous studies have examined the effects of pre-natal exposures on nAChRs in the brain or brainstem of autopsied infants and in relationship to SIDS (19, 22–25), including in the American Indian population, which is at a high risk for pre-natal exposure and SIDS death (21). These studies were, however, retrospective in nature with exposure information collected at autopsy. The strengths of the Safe Passage Study include its prospective design and rigorous assessment of quantity, frequency, and timing of pre-natal alcohol and smoking exposures. It is also uniquely focused on brainstem analysis in at-risk SIDS minority populations. Using cases collected from the Safe Passage Study and receptor ligand autoradiography, we tested the 2-fold hypothesis that (1) nAChR binding, as determined by binding to nAChR agonist ^125^I-epibatidine, is significantly altered in medullary centers and pontine sites related to cardiorespiratory function and arousal in SIDS infants compared to controls and (2) that pre-natal exposure to alcohol and smoking modifies ^125^I-epibatidine binding in these same brainstem sites.

## Materials and Methods

### Design of the Safe Passage Study

The study's hypotheses, specific aims, common protocol, enrollment, shipping, compliance, and specimen donation have been described in detail (34), as well as the approach to autopsy consent in socioeconomically disadvantaged populations (47). In brief, the Safe Passage Study was an international prospective, multicenter longitudinal cohort study with data collection conducted between August 2007 and October 2016. Clinical sites were selected based upon known high rates of maternal drinking and smoking during pregnancy and known high rates of SIDS in the population; however, all women from the catchment areas presenting for care at these sites were eligible to participate. These predominately include pregnant (1) American Indian and Caucasian women from the Northern Plains and (2) mixed ancestry women of the Western Cape, South Africa. Screening and enrollment occurred at pre-natal clinics affiliated with each clinical site between 6 weeks gestation up to, but not including, delivery. The maternal and fetal/infant dyads were followed during pregnancy and from birth until infants were 1 year of age, i.e., the risk period of SIDS. Detailed information regarding quantity, frequency, and timing of substance use was self-reported up to 4 times during pregnancy (recruitment, 20–24, 28–32, and 34+ gestational weeks) and at 1 month post-delivery. At sites in South Africa, a medico-legal autopsy was performed upon demise. Consent for research was sought from the family as soon as possible after death (47). At sites within the United States, an autopsy was regularly ordered by the coroner/medical examiner after which the family was approached for consent to the donation of tissue for research purposes. If consent was given, brain portions were frozen and shipped on dry ice to the Developmental Brain and Pathology Center (DBPC), Department of Pathology, Boston Children's Hospital, the centralized laboratory for research analysis (48). The study, including the use of brain tissue, was approved by the Institutional Review Boards (IRBs) of the local hospitals at which the infants were autopsied and Boston Children's Hospital. When designed, the Safe Passage Study for brain analysis anticipated and was powered on 37 SIDS cases and 37 non-SIDS controls, which would allow detection of a difference in receptor binding levels as small as 0.67 standard deviation.

### Clinical Database

SIDS was defined using a study definition that included the sudden unexpected death of an infant, <1 year of age, whose cause of death remained unexplained after review of all available information, including performance of a complete autopsy, examination or report of the death scene, and review of the clinical history (1). In addition, SIDS included deaths that might otherwise have been classified as undetermined including infants dying in unsafe sleep conditions but without evidence of mechanical asphyxia or suffocation by overlay. Known cause of death (KCOD) controls were defined as infants whose cause of death was identified after review of all available information (33). All demises were adjudicated by a team of pediatric pathologists, neuropathologists, forensic pathologists, a neonatologist, geneticist, obstetrician, and developmental psychologist who were blinded to pre-natal exposures. Case reviews did include toxicology, genetic testing (when appropriate and available), and metabolic testing (when appropriate and available). The postmortem interval (PMI) was calculated as the time when the infant was last seen alive to proclamation of time of discovery, as in previous studies by us (16, 49). Prospective collection of demise cases in the Safe Passage Study included 28 SIDS and 38 control cases that died after discharge from the hospital (post-discharge) (33). Of these cases, 12 SIDS and 10 post-discharge KCOD (post-KCOD) controls were available for neurochemical studies. There were 16 SIDS and 28 post-KCOD cases that were accrued in the Safe Passage Study but not available for neurochemistry either due to a lack of consent for autopsy research or due to technical issues related to quality of tissue. There were 45 KCOD controls that died prior to leaving the hospital after delivery [pre-discharge KCOD (pre-KCOD)] and 10 of these were analyzed for ^125^I-epibatidine binding to provide baseline developmental data.

### Brainstem Accrual

At autopsy, the brain was removed, weighed fresh, and examined for gross developmental and acquired abnormalities. The entire brainstem was removed from the level of the midbrain at the mammillary bodies to the cervicomedullary junction in 1.5-cm samples, sectioned on a Leica motorized cryostat at 20 μm, mounted on glass microscopic slides, and stored at −80°C until used for tissue receptor autoradiography. Frozen blocks were stored at −80°C in air-tight plastic containers.

### ^125^I-Epibatidine Binding and Generation of Brainstem Autoradiograms

To examine nicotinic receptors, we used receptor ligand autoradiography. Unlike homogenate radioligand binding, autoradiography allows the visualization of spatial anatomy and provides details of regional expression patterns (50). It is quantitative in nature and thus provides benefits over other techniques like immunohistochemistry. Radioligand ^125^I-epibatidine was used for autoradiographic analysis. Epibatidine is a nAChR agonist with high affinity to α4β2 nAChRs, one of the major subtypes of nAChRs in the brain, and with lower affinity to α7 nAChRs (51). The autoradiography procedures were performed according to the detailed methodology previously reported from our laboratory for ^3^H-epibatidine (52), with modification for the iodinated ligand. All steps were performed at room temperature. In brief, total ^125^I-epibatidine binding was determined by incubation of the frozen, unfixed sections with 0.5 nM ^125^I-epibatidine (2200 Ci/mmol, PerkinElmer, Waltham, MA, USA) in binding buffer consisting of 50 nM Tris–HCl, 120 mM NaCl, 5 mM KCl, 2.5 mM CaCl_2_, and 1 mM MgCl_2_, pH 7.4 for 60 min, an incubation time sufficient for equilibrium to be reached in epibatidine binding experiments (53). Non-specific binding was determined in adjacent sections by addition of 0.5 nM ^125^I-epibatidine and 300 μm of L-nicotine bitartrate. To remove unbound ligand, the sections were washed in a series of buffer changes (5 min each) followed by 3 dips in distilled water. Sections were then left overnight for drying, after which they were placed in cassettes and exposed to a BAS_TR2025 phosphoimaging plate (GE Healthcare Life Sciences, Marlborough, MA) for 20 h, with a set of ^125^I standards (American Radiolabeled Chemicals, Inc, MO, USA) calibrated by the manufacture in terms of radioactivity per unit weight. The standards allowed for the conversion of relative optical density to fentamoles per milligram (fmol/mg) of tissue to determine binding levels. A BAS-500 Bioimaging Analyzer (Fuji-Film) with Image Reader version 1.8 software (FujiFilm) was used to generate digital autoradiographic images from phosphoimaging plates. Quantitative densitometry of autoradiograms was performed using a MCID 5+ imaging system (Imaging Research). Specific receptor binding was determined by subtracting non-specific binding from total binding in individual tissue sections. The same sections incubated for autoradiography were subsequently stained with hematoxylin and eosin for anatomical assessment.

### Analysis of ^125^I-Epibaditine Binding in Homeostatic Brainstem Sites

**Figure 1:**
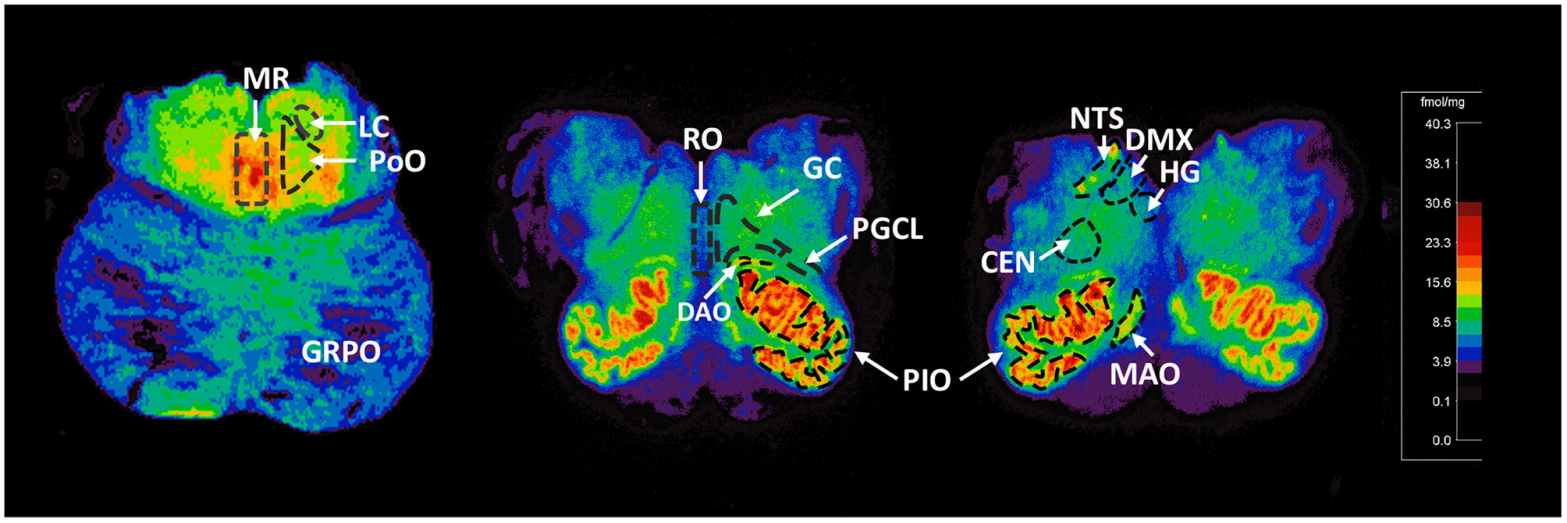
Representative distribution of ^125^I-epibatidine binding in the pons and medulla. Illustrative autoradiograms displaying ^125^I-epibatidine binding in tissue sections at the level of the pons (left), rostral medulla (middle), and mid medulla (right). Sections are taken from a 52-postconceptional week SIDS case (pons) and a 45-postconceptional week SIDS case (rostral and mid medulla) case. Receptor binding is normalized to the same scale (shown). The amount of receptor binding in fmol/mg is indicated with color according to the scale. The nuclei measured are denoted with dashed boundary lines and labeled. These nuclei include the following: (Pons) MR, median raphe; LC, locus coeruleus; PoO, nucleus pontis oralis; GRPO, griseum pontis; (Rostral Medulla) RO, raphe obscurus; GC, gigantocellularis; PGCL, paragigantocellularis lateralis; PIO, principal inferior olive; DAO, dorsal accessory olive; (mid medulla) HG, hypoglossal nucleus; DMX, dorsal motor nucleus of the vagus; NTS, nucleus of the solitary tract; CEN, centralis; MAO, medial accessory olive.

For relevance, we provide detail of the neuroanatomical functions and connectivity of the medullary and pontine nuclei studied here (see Supplemental Material). The human brainstem sites measured (Figure 1) were defined with reference to Olszewski and Baxter brainstem atlas (54) and confirmed with Paxinos and Huang brainstem atlas (55). For each case, ^125^I-epibatdine binding in the brainstem was measured at 3 levels; mid medulla at the level of nucleus of Roller, which included the nucleus of the solitary tract (NTS) (all visceral sensory inputs of the autonomic nervous system and sympathetic autonomic system integration), the hypoglossal nucleus (HG) (airway patency, especially during sleep), dorsal motor nucleus of the vagus (DMX) (preganglionic vagal outflow of the parasympathetic autonomic nervous system), centralis (CEN), principal inferior olive (PIO), and medial accessory olive (MAO) (the cerebellar network); rostral medulla at the level of the nucleus pre-positus, which included the raphe obscurus (RO), gigantocellularis (GC), paragigantocellularis lateralis (PGCL), core nuclei of serotonergic homeostatic medullary network, and the dorsal accessory olive (DAO); and rostral pons at the level of nucleus parabrachialis lateralis, which included the locus coeruleus (LC) (major source neurons of the noradrenergic ascending arousal network), nucleus pontis oralis (PoO) (part of source neurons of the cholinergic ascending arousal system), griseum pontis (GRPo) (pre-cerebellar nucleus, part of the pontocerebellar network), and median raphe (MR) (part of the rostral 5-HT ascending arousal network) (Figure 1). With the exception of the midline nuclei (RO and MR), ^125^I-epibatidine binding was measured from both sides (left and right) of the section and the means calculated to determine final value in fmol/mg.

### Collection of Exposure Data

This study used a modified timeline follow-back method to collect exposure related to maternal smoking/drinking during pregnancy (56). At each visit during and after pregnancy, the participant was asked about the last date of use (separately for alcohol and smoking). For data relating to alcohol, they were asked about consumption for ±15 days around last menstrual period, as well as 30 days prior to the last drinking day since their last research appointment. For smoking, they were asked about the frequency of smoking and number of cigarettes on a typical day for the 30 days prior to the last date of use since their last research appointment. To estimate the total number of drinks consumed during pregnancy, each drink consumed was first standardized where 1 drink is defined as 14 g of ethanol. The study design did not allow consumption data to be collected on every single day of pregnancy, so missing values were imputed using the k-nearest neighbor (kNN) method. Methods for alcohol imputation are cited elsewhere (57). Since frequency of cigarette use was collected more sparsely, average cigarettes smoked per week during pregnancy was used. The number of cigarettes smoked each week during pregnancy was calculated, and missing weeks was imputed in a similar way to the alcohol imputation. After imputation, an average number of cigarettes during pregnancy was calculated.

### Statistical Analysis

Descriptive analysis was conducted to analyze differences between cause of death (SIDS vs. KCOD controls) and demographics, maternal substance use during pregnancy, infant sleep practices, and relevant autopsy and clinical findings. Analyses used included Student's *t*-test or Mann–Whitney *U* for continuous variables and chi-square testing with Fisher's exact test for categorical variables. Maternal demographics assessed included age, education, housing type, history of loss by SIDS, and delivery type. Infant demographics assessed included birth weight and length, gestational age at birth, post-natal age at death, gender, and race. Autopsy findings assessed included postmortem interval, body weight at autopsy, and brain weight at autopsy. Maternal use of alcohol and smoking during pregnancy were assessed as binary values (used during pregnancy or not) and as continuous values (total number of drinks during pregnancy and average number of cigarettes per week). Maternal use of alcohol and smoking by trimester was assessed as continuous values (number of drinks by trimester and average cigarettes per week). Infant sleep practices assessed included sleep position last placed, sleep position found, and whether or not the infant was covered by bedding or blankets.

Multivariate linear regression models were built to analyze differences in mean ^125^I-epibatidine binding values by case diagnosis and exposure. Post-conceptional age (PCA) was controlled for in all models, as it is significantly different by case diagnosis and associated with ^125^I-epibatidine binding. A subanalysis assessed the effect of development (PCA) on ^125^I-epibatidine binding in the 10 pre-KCOD- and 10 post-KCOD-control infants. There was no effect of PMI on binding; therefore, PMI was not controlled for in the analyses. *p* < 0.05 were considered statistically significant. Analyses were conducted using SAS 9.4.

## Results

### Clinicopathological Information

The demise cohort for ^125^I-epibatidine analyses include SIDS (*n* = 12), post-KCOD controls (*n* = 10), and pre-KCOD controls (*n* = 10) (see above). The causes of death for the control groups are given in Table 1, with demises separated as pre- or post-discharge, based on whether the infant died in the hospital without being discharged after birth or at home after discharge. Selected demographic data including incidence of exposure are summarized in Table 2. Pre-KCOD cases were included only for the purpose of looking at developmental changes in receptor binding. Pre-KCOD cases (*n* = 10) ranged from 25.8 to 41.9 postconceptional weeks (mean = 32.8 weeks) (Table 2). Sixty percent of pre-KCOD cases were male (*n* = 6) and 80% (*n* = 8) were South African mixed race with the other 20% being American Indian (*n* = 1) or Caucasian (*n* = 1). The SIDS cohort was statistically analyzed relative to the post-KCOD cases only. In the comparison between SIDS and post-KCOD, there was no significant difference in mean gestational age (GA), PCA, PMI, birth weight, sex, pre-mature birth, autopsy body, or brain weight (Table 2). The majority of cases was from the South Africa clinical site (Table 2), accounting for the predominately South African mixed-race assignment in SIDS (92%) and post-KCOD (80%) [*p* = non-significant (ns)]. We assessed several maternal characteristics and found a significant difference only in education status (*p* = 0.03) with the majority of post-KCOD mothers completing high school (60%) and the majority of SIDS mothers reporting some high school education (67%) (Table 2). Other demographic measures were not significantly different between SIDS and post-KCOD controls. These include crowding index (>1 person/room), employed (yes/no), marital status, and housing type (council housing, informal shack/squatter, apartment/house, other) (data not shown).

**Table 1:** Causes of death in pre- and post-discharge known cause of death (KCOD) cases.

**Case**	**Pre- or Post-discharge**	**GA (wks)**	**PNA (wks)**	**PCA (wks)**	**Cause of death**
1	Pre-discharge	25.7	0.1	25.8	Complications of pre-maturity
2	Pre-discharge	27.0	0.04	27.0	Hyaline membrane disease, chorioamnionitis and placental abruption
3	Pre-discharge	27.0	0.6	27.6	Pre-eclampsia and pre-maturity
4	Pre-discharge	30.3	1.4	31.7	Omphalocele, peritonitis, sepsis
5	Pre-discharge	32.6	0.1	32.7	Pulmonary hemorrhage
6	Pre-discharge	32.9	0.1	33.0	Fetal head trauma due to motor vehicle accident
7	Pre-discharge	31.7	1.6	33.3	Klebsiella pneumonia, necrotizing enterocolitis, jaundice
8	Pre-discharge	37.0	0.04	37.0	Intrauterine growth restriction
9	Pre-discharge	37.5	0.02	37.5	Pulmonary hypoplasia, multicystic dysplastic kidney disease complicating Potter's sequence
10	Pre-discharge	41.3	0.6	41.9	Meconium aspiration, severe bronchopneumonia, perinatal asphyxia
11	Post-discharge	27.3	9.4	36.7	Respiratory infection
12	Post-discharge	35.6	2.1	38.0	Respiratory infection
13	Post-discharge	27.6	12.9	40.5	CNS infection
14	Post-discharge	38.9	1.6	40.5	Congenital defects
15	Post-discharge	32.1	12.1	44.2	Renal; tubule-interstitial nephritis
16	Post-discharge	36.0	9.0	45.0	Respiratory infection
17	Post-discharge	40.0	11.7	51.7	Respiratory infection
18	Post-discharge	38.3	14.1	52.4	CNS infection
19	Post-discharge	39.6	22.7	62.3	Gastrointestinal infection
20	Post-discharge	38.6	25.9	64.4	Respiratory infection

*Pre-discharge cases are infants who died in the hospital prior to being released after birth. Post-discharge cases are infants that died after discharge from the hospital. KCOD, known cause of death; CNS, central nervous system; N, number; GA, gestational age; PNA, post-natal age; PCA, postconceptional age; wks, weeks*.

**Table 2:** Selected demographic information.

	**Pre-discharge Pre-KCOD**		**Post-discharge**				
			**SIDS**		**Post-KCOD**		***p-*value**
	***N***	**Mean ± STD or *n* (%) or median**	***N***	**Mean ± STD or *n* (%) or median**	***N***	**Mean ± STD or *n* (%) or median**	
Maternal age (yrs)	10	25.8 ± 7.8	12	27.1 ± 4.9	10	27.0 ± 6.1	0.97
Maternal history of loss by SIDS	6	0 (0)	11	1 (9)	7	0 (0)	1.00
Caesarian section	10	5 (50)	12	3 (25)	10	3 (30)	1.00
Maternal education	9		12		10		**0.03**
Any primary school		0 (0)		2 (17)		0 (0)	
Some HS		7 (78)		8 (67)		4 (40)	
Completed HS		2 (22)		1 (8)		6 (60)	
Beyond HS		0 (0)		1 (8)		0 (0)	
GA (wks)	10	32.3 ± 5.1	12	36.1 ± 3.4	10	35.4 ± 4.8	0.70
PCA (wks)	10	32.8 ± 5.1	12	49.0 ± 12.1	10	47.6 ± 9.8	0.76
PMI (h)	10	44.1 ± 34.4	12	41.6 ± 25.3	10	47.6 ± 37.1	0.66
Birth weight (g)	10	1,590.8 ± 803.4	12	2,388.3 ± 785.0	10	2,535.0 ± 1,061.0	0.71
Birth length (cm)	2	33.5 ± 3.5	10	47.4 ± 4.3	8	47.8 ± 5.7	0.88
Male sex	10	6 (60)	12	6 (50)	10	6 (60)	0.69
Pre-term birth (<37 GA wks)	10	7 (70)	12	7 (58)	10	5 (50)	1.0
Race	10		12		10		0.71
American Indian		1 (10)		0 (0)		1 (10)	
South African mixed race		8 (80)		11 (92)		8 (80)	
Caucasian		1 (10)		1 (8)		1 (10)	
Autopsy body wt (g)	10	2,283.3 ± 1,723.7	12	4„168.7 ± 1,935.7	9	3672.4 ± 1,870.0	0.56
Autopsy brain wt (g)	9	236.2 ± 126.9	10	510.3 ± 173.9	6	507.8 ± 238.1	0.98
Occipital frontal çircum (cm)	10	29.0 ± 4.9	12	37.4 ± 4.6	9	37.5 ± 4.9	0.97
Alcohol during pregnancy (y/n)	8	5 (63)	12	6 (50)	10	7 (70)	0.41
*N* drinks in pregnancy	10	0.9	12	2.6	10	8.3	0.48
Smoking during pregnancy (y/n)	8	8 (100)	12	12 (100)	10	9 (90)	0.45
Avg cigarettes/week	6	9.3	11	20.6	8	28.7	0.90

*N represents the number of cases with available demographic information. KCOD, known cause of death; SIDS, sudden infant death syndrome; yrs, years; HS, high school; GA, gestational age; PCA, postconceptional age; PMI, postmortem interval; wks, weeks; hrs, hours; circum; circumference; cm, centimeter; y/n, yes/no; Avg, average; g, grams, STD, standard deviation. Significant p < 0.05 are in bold*.

The presence of infection in KCOD controls, including peripheral and central infection, is noted in Table 1. Evidence of mild infection was noted at autopsy in 7 out of 12 SIDS cases including group B *Streptococcus* in the lungs (*n* = 1), inflammatory changes in the larynx (*n* = 1), inflammatory changes in the pharynx (*n* = 1), inflammatory cells in the lamina propria (*n* = 1), mild pneumonitis (*n* = 2), and positive *Clostridioides difficile* in the stool (*n* = 1).

Information regarding maternal smoking and drinking during pregnancy was available for all cases in the SIDS (*n* = 12) and post-KCOD cases (*n* = 10). The incidence of maternal smoking during pregnancy (yes or no) was 100% (12/12) in the SIDS group (Table 2) and ranged from an average of 0.1 cigarettes per week to 62.3 (median of 20.6) (Table 3A). This was not statistically different from post-KCOD cases whose incidence of smoking was 90% (Table 2) and ranged from an average of 0 cigarettes per week to 58.6 (median of 28.7) (Table 3A). Maternal smoking was neither statistically different between SIDS and controls in any one trimester nor was it statistically different across trimesters in SIDS or in controls. The incidence of maternal drinking during pregnancy (yes or no) was 50% (6/12) in the SIDS group (Table 2) and ranged from 0 drinks during pregnancy to 210.75 drinks in pregnancy (median of 2.6) (Table 3B). This was not statistically different from post-KCOD cases whose incidence of drinking was 70% (Table 2) and ranged from 0 drinks during pregnancy to 102.5 drinks in pregnancy (median of 8.3) (Table 3). There was no statistical difference between SIDS and post-KCOD cases when exposure was analyzed by trimesters (Tables 3A,B).

**Table 3A:** Smoking through pregnancy and by trimesters.

	***N***	**Mean**	**STD**	**Median**	**Min**	**Max**	**Wilcoxin *p*-value**
**Through pregnancy**							
Average cigarettes/week							0.90
SIDS	11	27.1	20.14	20.6	0.1	62.3	
Post-KCOD	8	26.8	23.91	28.7	0	58.6	
**By trimester**							
**Average cigarettes/week by trimester**							
Trimester 1							0.54
SIDS	11	26.7	20.06	23.7	0	61.7	
Post-KCOD	8	19.2	20.32	12.1	0	52.4	
Trimester 2							0.90
SIDS	11	27.9	21.63	22.5	0	64.4	
Post-KCOD	8	27.7	23.80	27.6	0	60.0	
Trimester 3							0.72
SIDS	11	26.8	19.59	21.3	0.2	60.4	
Post-KCOD	7	27.7	31.79	7.7	0	67.6	

*N, number; STD, standard deviation; Min, minimum; Max, maximum; Post-KCOD, post-discharge known cause of death; SIDS, sudden infant death syndrome*.

**Table 3B:** Drinking exposure through pregnancy and by trimesters.

	***N***	**Mean**	**STD**	**Median**	**Min**	**Max**	**Wilcoxin *p*-value**
**Through pregnancy**							
*N* drinks in pregnancy							0.48
SIDS	12	25.1	59.57	2.6	0	210.75	
Post-KCOD	10	25.3	35.42	8.3	0	102.5	
**By trimester**							
***N*** **drinks by trimester**							
Trimester 1							0.23
SIDS	12	8.3	22.80	0	0	80.1	
Post-KCOD	10	10.1	13.56	3.6	0	37.5	
Trimester 2							0.65
SIDS	12	13.6	27.57	0	0	94.8	
Post-KCOD	10	13.5	28.27	0	0	69.2	
Trimester 3							0.96
SIDS	12	3.3	10.31	0	0	35.9	
Post-KCOD	10	1.6	4.55	0	0	14.5	

*N, number; STD, standard deviation; Min, minimum; Max, maximum; Post-KCOD, post-discharge known cause of death; SIDS, sudden infant death syndrome*.

Information regarding sleep-related risk factors was available for all cases within the SIDS group but only 2 of the 10 post-KCOD controls (data not shown). Within the SIDS group, the prevalence of infants last placed supine was 8% (1/12), on their side (lateral) was 33% (4/12), and prone was 58% (7/12). The prevalence of SIDS infants in the demised state on their side (lateral) was 64% (7/11) and in the prone position was 36% (4/11). In the post-KCOD group, the position last being placed supine was two of two; one of two was found dead in the supine position, and the other was found prone.

Information regarding post-natal exposure to cigarette smoke was available in 11 out of 22 (50%) of post-discharge infants. Of these 11, only 5 (2 SIDS and 3 post-KCOD controls) reported the infant being exposed to cigarettes at home. Information regarding other illicit drugs (i.e., marijuana and methamphetamine) was available on 24 of 32 total KCOD controls and SIDS. In total, only 4 cases (one pre-KCOD control, two post-KCOD controls, and one SIDS) reported any illicit drug use.

### ^125^I-Epibatidine Binding Distribution in the Developing Brainstem

**Figure 2:**
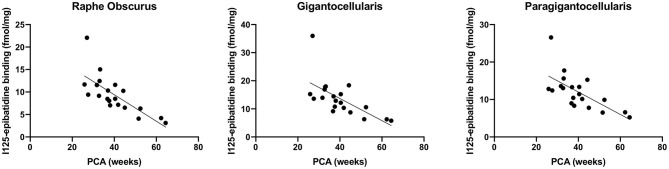
Developmental decrease in ^125^I-epibatidine binding in the rostral medulla. Combined pre- and post-KCOD controls show a decrease in ^125^I-epibatidine binding in nuclei of the rostral medulla. This relationship is illustrated in the RO (*p* = 0.0002), GC (*p* = 0.002), and PGCL (*p* = 0.001).

We assessed ^125^I-epibatidine binding in the medulla and the pons from 26 postconceptional (PC) weeks (midgestation) to 64 PC weeks (~6 post-natal months) in the pre-KCOD (n = 10) and post-KCOD (*n* = 10) cases for developmental information. The inclusion of pre-KCOD controls allowed us to analyze a greater range of ages and provide increased information on development through gestation. By midgestation binding was differentially localized to nuclei of interest in a relatively fixed distribution (Figure 1). Medullary binding patterns across all controls combined showed the highest binding in the PIO and lowest binding in the RO (Table 4, Figure 1). In the rostral pons, binding in nuclei of the pontine tegmentum (MR, LC, and PoO) were relatively high compared to measurements in the basis pontis (GRPO) (Table 4, Figure 1). There was a significant developmental decrease in binding with increasing postconceptional age (*p* < 0.05) in the RO, GC, PGCL, and DAO (beta values from −0.39 to −0.25) of the rostral medulla and in the CEN of the mid medulla (beta = −0.22) (Table 4). There was a marginally significant developmental decrease binding in in the LC and PoO of the rostral pons (*p* = 0.07; beta = −0.22 and *p* = 0.05; beta = −0.24, respectively). To illustrate this effect, a decrease in ^125^I-epibatidine binding with increasing PC age is shown for the RO, GC, and PGCL of the rostral medulla (Figure 2). Of note, three rostral medullary sites found to have decreased binding with age (RO, GC, and PGCL) were also found to be significantly affected by nicotine exposure when SIDS and post-KCOD controls were combined (see **Table 6**). The effects of age on these sites remained significant even after adjusting for the effect of smoking (see **Table 6**).

**Table 4:** Effect of PCA in Pre- and Post-KCOD controls combined on ^125^I-epibatidine binding in brainstem nuclei.

	**PCA-adjusted mean of** ^****125****^**I-epibatidine binding in specific brainstem nuclei**		**Effect of PCA on** ^****125****^**I-epibatidine binding**	
	***N***	**Mean ± SE (fmol/mg)**	**Beta**	***p*-value**
**Mid medulla**				
HG	20	13.03 ± 2.42	−0.21	0.37
DMX	20	13.36 ± 1.69	0.19	0.26
NTS	20	15.22 ± 2.53	0.21	0.39
CEN	20	11.82 ± 1.02	−0.22	**0.03**
PIO	20	23.46 ± 3.08	0.27	0.37
MAO	19	16.58 ± 1.84	−0.01	0.94
**Rostral medulla**				
RO	20	9.35 ± 0.65	−0.29	**0.0002**
GC	20	13.63 ± 1.15	−0.39	**0.002**
PGCL	20	11.95 ± 0.81	−0.30	**0.001**
DAO	16	14.71 ± 1.05	−0.25	**0.022**
PIO	20	16.82 ± 0.97	0.06	0.53
**Rostral pons**				
MR	16	15.75 ± 1.47	−0.22	0.13
LC	16	13.46 ± 1.20	−0.22	*0.07*
PoO	16	14.24 ± 1.22	−0.24	*0.05*
GRPO	16	6.19 ± 0.79	0.01	0.89

*Significant p (<0.05) are bolded. Marginal p (> 0.05 and <0.1) are in italics*.

*The beta values represent the degree of change in ^125^I-epibatidine binding associated with a change in postconceptional age (PCA). Negative beta values indicate a decrease in ^125^I-epibatidine binding with PCA. All binding values are in fmol/mg. N, number of cases measured; SE, standard error. Pre-KCOD, predischarge known cause of death; Post-KCOD, post-discharge KCOD; HG, hypoglossal nucleus; DMX, dorsal motor nucleus of the vagus; NTS, nucleus of the solitary tract; CEN, centralis; PIO, principal inferior olive; MAO, medial accessory olive; RO, raphe obscurus; GC, gigantocellularis; PGCL, paragigantocellularis lateralis; DAO, dorsal accessory olive; MR, median raphe; LC, locus coeruleus; PoO, nucleus pontis oralis; GRPo, griseum pontis*.

### ^125^I-Epibatidine Binding Between All SIDS and All Post-KCOD Controls

Using analysis of covariance (ACOVA) controlling for the effect of PCA, there was a significant effect of diagnosis only in rostral pons PoO (*p* = 0.02) with post-KCOD controls having a higher binding than SIDS (12.1 ± 0.9 fmol/mg and 9.1 ± 0.8 fmol/mg, respectively) (Table 5). There was a marginally significant effect in the LC of the rostral pons (p = 0.08) with post-KCOD controls having a higher binding than SIDS (Table 5). The demise cohort within PASS has a relatively high prevalence of pre-term birth (58%, SIDS; 50% KCOD) (Table 2). Thus, we analyzed ^125^I-epibatidine binding in the PASS cohort based on pre-term (<37 gestational weeks) or term birth (>37 gestational weeks). ^125^I-Epibatidine binding from pre-term SIDS (*n* = 7) was compared to pre-term post-KCOD controls (*n* = 5), adjusting for PCA. Similarly, ^125^I-epibatidine binding from term SIDS (*n* = 5) was compared to term post-KCOD controls (*n* = 5), adjusting for PCA. We found no significant effect of diagnosis in any nuclei in either pre-term or term cases (data not shown).

**Table 5:** Effect of diagnosis controlling for PCA on ^125^I-epibatidine binding in the brainstem.

	**Cause of death**					**PCA**	
	**SIDS**		**Post-KCOD**				
	***N***	**Mean ± SE (fmol/mg)**	***N***	**Mean ± SE (fmol/mg)**	***p*-value**	**Beta**	***p*-value**
**Mid medulla**							
HG	12	8.3 ± 2.34	10	12.3 ± 2.56	0.26	−0.15	0.37
DMX	12	10.8 ± 2.32	10	14.2 ± 2.53	0.33	0.16	0.33
NTS	12	13.5 ± 3.70	10	17.0 ± 4.04	0.53	0.12	0.65
CEN	12	9.0 ± 1.26	10	9.3 ± 1.37	0.88	−0.23	**0.02**
PIO	12	19.8 ± 4.42	10	27.4 ± 4.83	0.27	−0.21	0.51
MAO	12	14.2 ± 3.13	10	16.1 ± 3.42	0.70	−0.31	0.17
**Rostral medulla**							
RO	12	6.8 ± 0.80	10	6.9 ± 0.87	0.94	−0.24	**<0.001**
GC	12	9.9 ± 1.11	10	10.4 ± 1.21	0.73	−0.30	**0.001**
PGCL	12	9.6 ± 1.08	10	9.2 ± 1.18	0.83	−0.27	**0.002**
DAO	9	13.6 ± 1.85	10	12.7 ± 1.67	0.72	−0.35	**0.011**
PIO	12	15.5 ± 1.34	10	17.9 ± 1.46	0.24	−0.02	0.82
**Rostral pons**							
MR	9	10.2 ± 1.55	7	13.0 ± 1.72	0.25	−0.02	0.88
LC	10	8.9 ± 0.89	7	11.5 ± 1.04	*0.08*	−0.11	*0.10*
PoO	10	9.1 ± 0.78	7	12.1 ± 0.91	**0.02**	−0.15	**0.02**
GRPO	10	5.9 ± 0.59	7	6.5 ± 0.69	0.53	−0.05	0.25

*Diagnosis p-values are adjusted for postconceptional age (PCA). Means estimated for PCA = 48. Significant p (<0.05) are bolded. Marginally significant (> 0.05 and <0.10) p-values are in italics. SE, standard error; Post-KCOD, post-discharge known cause of death; SIDS, sudden infant death syndrome; HG, hypoglossal nucleus; DMX, dorsal motor nucleus of the vagus; NTS, nucleus of the solitary tract; CEN, centralis; PIO, principal inferior olive; MAO, medial accessory olive; RO, raphe obscurus; GC, gigantocellularis; PGCL, paragigantocellularis lateralis; DAO, dorsal accessory olive; MR, median raphe; LC, locus coeruleus; PoO, nucleus pontis oralis; GRPo, griseum pontis*.

### Effect of Exposure on ^125^I-Epibatidine Binding

We assessed the effect of the amount of maternal drinking (number of drinks consumed) on ^125^I-epibatidine binding in all SIDS (*n* = 12) and post-KCOD controls (*n* = 10) combined, adjusting for PCA. There were no significant effects of amount of drinking on ^125^I-epibatidine binding in any nuclei (Table 6). Given that 50% of SIDS mothers and 70% of post-KCOD mothers reported drinking during pregnancy, we repeated the analyses with exposed-only, SIDS (*n* = 6), and post-KCOD controls (*n* = 7) combined. Similar to the analyses in Table 5, the analysis in alcohol exposed-only cases showed no effect of amount of drinking on ^125^I-epibatidine binding in any nuclei (data not shown). There was no effect of maternal drinking on pre-KCOD controls (data not shown). Similarly, we assessed the effects of amount of smoking on SIDS and post-KCOD controls combined, adjusting for PCA. A significant positive association between average cigarettes per week and ^125^I-epibatidine binding was found in the following rostral medullary nuclei: RO, GC, and PGCL (*p* = 0.01, 0.02, and 0.002, respectively) (Table 6). Given the effect of smoking on both SIDS and post-KCOD controls combined, we analyzed each group separately to determine if cigarette smoking had a differential effect on one compared to the other. In post-KCOD controls, we found a significant positive association between ^125^I-epibatidine binding and cigarettes smoked per week in the RO (*p* = 0.047) and the PGCL (*p* = 0.03) but little effect in any nuclei in SIDS (Table 7). Of note, there was a significant effect of PCA in the RO, GC, and PGCL of post-KCOD controls consistent with the developmental data of Table 4. Likewise, there was a significant effect of PCA in these same nuclei in SIDS cases. Given the effect of smoking on ^125^I-epibatidine binding in sites of the rostral medulla, we reanalyzed the SIDS vs. post-KCOD control data to examine the effect of diagnosis controlled for smoking and PCA. The significant effect of diagnosis remained in the PoO (*p* = 0.04). A marginal significance of diagnosis was seen in the GC (*p* = 0.07) and DAO (*p* = 0.07) (data not shown).

**Table 6:** Effects of exposure on ^125^I-epibatidine binding in the brainstem in SIDS and Post-KCOD controls combined.

		**Alcohol on** ^****125****^**I-epibatidine**					**Smoking on** ^****125****^**I-epibatidine**			
		***N*** **drinks per pregnancy**		**PCA**			**Ave. cigarettes per week**		**PCA**	
	***N***	**Beta**	***p*-value**	**Beta**	***p*-value**	***N***	**Beta**	***p*-value**	**Beta**	***p*-value**
**Mid medulla**										
HG	22	−0.01	0.76	−0.17	0.32	19	0.15	0.12	−0.19	0.31
DMX	22	−0.04	0.31	0.11	0.50	19	−0.04	0.56	0.15	0.27
NTS	22	−0.07	0.23	0.04	0.87	19	−0.11	0.30	0.10	0.64
CEN	22	−0.02	0.28	−0.25	**0.008**	19	0.04	0.15	−0.18	**0.006**
PIO	22	−0.05	0.50	−0.28	0.39	19	0.08	0.46	0.08	0.70
MAO	22	−0.05	0.32	−0.36	0.11	19	0.03	0.58	−0.12	0.34
**Rostral medulla**										
RO	22	−0.01	0.45	−0.25	**0.0003**	19	0.06	**0.01**	−0.19	**0.0001**
GC	22	−0.02	0.22	−0.32	**0.0005**	19	0.07	**0.02**	−0.25	**0.0002**
PGCL	22	−0.01	0.41	−0.28	**0.0014**	19	0.07	**0.002**	−0.20	**<0.0001**
DAO	19	−0.04	0.32	−0.35	**0.0074**	16	0.03	0.39	−0.20	**0.007**
PIO	22	−0.03	0.18	−0.06	0.54	19	0.01	0.80	0.001	1.00
**Rostral pons**										
MR	16	−0.004	0.93	0.004	0.97	15	−0.06	0.32	0.03	0.82
LC	17	0.01	0.85	−0.12	0.12	16	0.02	0.65	−0.11	0.15
PoO	17	0.02	0.52	−0.15	**0.04**	16	0.03	0.45	−0.16	**0.04**
GRPO	17	−0.03	0.10	−0.07	0.10	16	0.02	0.42	−0.08	*0.09*

*Significant p (<0.05) are bolded. Marginal p (> 0.05 and <0.1) are in italics. The effects of individual exposure (N drinks per pregnancy or average cigarettes per week) on ^125^I-epibatidine binding is adjusted for PCA. N, number; PCA, postconceptional age in weeks, Ave, average; Post-KCOD, post-discharge known cause of death; SIDS, sudden infant death syndrome; HG, hypoglossal nucleus; DMX, dorsal motor nucleus of the vagus; NTS, nucleus of the solitary tract; CEN, centralis; PIO, principal inferior olive; MAO, medial accessory olive; RO, raphe obscurus; GC, gigantocellularis; PGCL, paragigantocellularis lateralis; DAO, dorsal accessory olive; MR, median raphe; LC, locus coeruleus; PoO, nucleus pontis oralis; GRPo, griseum pontis*.

**Table 7:** Effects of maternal cigarette smoking on ^125^I-epibatidine binding in the brainstem in SIDS and Post-KCOD.

		**Smoking on epibatidine—SIDS Only**					**Smoking on epibatidine—Post-KCOD Only**			
	***N***	**Ave. cigarettes per week**		**PCA**		***N***	**Ave. cigarettes per week**		**PCA**	
		**Beta**	***p*-value**	**Beta**	***p*-value**		**Beta**	***p*-value**	**Beta**	***p*-value**
**Mid medulla**										
HG	11	0.07	0.22	−0.11	0.24	8	0.23	0.33	−0.19	0.71
DMX	11	−0.06	0.50	0.13	0.38	8	−0.01	0.93	0.22	0.48
NTS	11	−0.27	*0.07*	0.23	0.34	8	0.03	0.87	0.01	0.98
CEN	11	0.04	0.30	−0.19	**0.02**	8	0.05	0.41	−0.14	0.30
PIO	11	−0.08	0.21	0.22	*0.07*	8	0.23	0.34	0.06	0.91
MAO	11	−0.004	0.93	−0.12	0.21	8	0.08	0.55	−0.02	0.95
**Rostral medulla**										
RO	11	0.05	0.18	−0.17	**0.02**	8	0.06	**0.047**	−0.21	**0.01**
GC	11	0.05	0.13	−0.20	**0.006**	8	0.09	0.10	−0.31	**0.03**
PGCL	11	0.05	0.10	−0.19	**0.004**	8	0.09	**0.03**	−0.19	**0.04**
DAO	8	−0.03	0.45	−0.06	0.44	8	0.03	0.59	−0.19	0.16
PIO	11	−0.11	*0.10*	0.14	0.20	8	0.12	0.17	−0.12	0.53
**Rostral pons**										
MR	9	−0.05	0.63	0.04	0.80	6	−0.10	0.41	−0.07	0.82
LC	10	0.03	0.64	−0.11	0.30	6	−0.01	0.85	−0.16	0.31
PoO	10	0.04	0.37	−0.15	*0.06*	6	−0.01	0.93	−0.23	0.27
GRPO	10	0.01	0.67	−0.05	0.21	6	0.01	0.91	−0.15	0.35

*Significant p (<0.05) are bolded. Marginally significant p (> 0.05 and <0.1) are in italics. PCA, postconceptional age; Ave, average; Post-KCOD, post-discharge known cause of death; SIDS, sudden infant death syndrome; HG, hypoglossal nucleus; DMX, dorsal motor nucleus of the vagus; NTS, nucleus of the solitary tract; CEN, centralis; PIO, principal inferior olive; MAO, medial accessory olive; RO, raphe obscurus; GC, gigantocellularis; PGCL, paragigantocellularis lateralis; DAO, dorsal accessory olive; MR, median raphe; LC, locus coeruleus; PoO, nucleus pontis oralis; GRPo, griseum pontis*.

## Discussion

The Safe Passage Study is the first prospective, multicenter longitudinal study to provide evidence that infants exposed to pre-natal alcohol and cigarette smoke continuing beyond the first trimester have substantially higher risk of SIDS, as compared to those unexposed or exposed only in the first trimester (33). While the first trimester is critical for neurulation and neurogenesis, brain development through the second and third trimesters involves neuronal maturation, synaptogenesis, and synaptic reorganization and pruning—processes that are adversely affected by exposure to nicotine and alcohol (58). Although the fundamental mechanism of pre-natal exposures upon SIDS risk is unknown, we hypothesize that it involves adverse effects on the cholinergic receptor system during brain development. The Safe Passage Study provided an opportunity to examine this hypothesis in an international cohort of prospectively collected cases. Our major findings are as follows: (1) there is a developmental decrease in ^125^I-epibatidine binding with age in 5 sites within the rostral and mid medulla; (2) there is a decrease in ^125^I-epibatidine binding in the PoO, a critical component of the cholinergic ascending arousal system of the rostral pons, in SIDS compared to post-KCOD controls; and (3) smoking affected ^125^I-epibatidine binding in 3 rostral medullary sites that contain 5-HT neurons and that have been shown to be abnormal in SIDS infants when examined for the serotonin receptor 1A (5-HT_1A_) (16, 49). These new prospective analyses not only provide reproducibility of developmental changes in cholinergic systems within the brainstem and select cholinergic abnormalities in SIDS but also provide new insights and hypotheses regarding mechanisms by which pre-natal exposures may adversely affect critical medullary neurotransmitter systems involved in cardiorespiratory functions.

### ^125^I-Epibatidine Binding in the Brainstem—Effect of PCA on KCOD Controls

The baseline (control) expression pattern of the nicotinic receptors in the brainstem of humans and other species has been studied extensively at the mRNA, protein, and receptor level via the methods of *in situ* hybridization, immunohistochemistry, and receptor-ligand binding autoradiography, respectively [reviewed in Vivekanandarajah et al. (36)]. The relative distribution of ^125^I-epibatidine binding in selected human infant brainstem sites of this study is similar in pattern to previous observations with ^3^H-epibatidine binding (40), ^3^H-nicotine binding (19, 21, 59), and immunohistochemistry (24, 60). In this study, we observed a significant decrease in ^125^I-epibatidine binding with increasing PCA in all controls combined (pre- and post-discharge) in the rostral medullary nuclei RO, GC, PGCL, and DAO, mid-medullary nucleus CEN, and a marginally significant effect of age in the PoO of the rostral pons. Given that our KCOD controls ranged from 26 to 64 PC weeks, these data provide information on nAChRs and epibatidine binding prenatally through the second half of gestation and postnatally into the first 6 months of life. The decrease in binding with age suggests that this developmental window is a dynamic period of cholinergic influence on brain development and function. While a decrease in the DAO, PoO, and CEN with age has previously been reported using either ^3^H-epibatidine (DAO) (40) or ^3^H-nicotine (CEN, PoO) (19), a developmental decrease in the RO, GC, and PGCL using ^125^I-epibatidine as a ligand has not been shown. The RO, GC, and PGCL nuclei of the medullary 5-HT system contain 5-HT cells and project diffusely to other regions of the brainstem and the spinal cord. These nuclei form part of the homeostatic network of the rostral medulla, critically involved in cardiorespiratory integration and arousal (11). Serotonergic neurons within the nuclei mediate these homeostatic responses (11, 61, 62). Given that serotonergic neurons in these regions express nicotinic receptors (40) and that these serotonergic neurons are likely undergoing developmental changes in 5-HT neurotransmission (as determined by developmental changes in ligand binding to general 5-HT receptor agonist ^3^H-LSD), particularly from midgestation to infancy (63), developmental changes in ^125^I-epibatidine binding support a dynamic and complex relationship between the neurotransmitter systems—a relationship likely vulnerable to dysregulation during development due to pre-natal exposure to nicotine and inappropriate nicotinic receptor binding at these sites. Evidence supporting the effects of nicotine on 5-HT function is detailed below. Studies of additional neurotransmitter systems (including 5-HT) in the PASS cohort are warranted to address potential interrelationships between receptor systems.

### ^125^I-Epibatidine Binding Between SIDS and Post-KCOD Controls

In our analysis between SIDS and post-KCOD controls, we found no difference in ^125^I-epibatidine binding in the SIDS brainstem in 14 out of 15 brainstem sites. This lack of difference is consistent with the findings of Duncan et al. (21) and Nachmanoff et al. (19), both of which used nicotine as a ligand as opposed to epibatidine and both of which showed no difference in any nuclei when SIDS were compared to controls. We did, however, find a significant decrease in SIDS compared to post-KCOD controls in the PoO and a marginal decrease in the LC, both nuclei of the rostral pons. The LC and PoO are components of the extrathalamic and thalamic arousal system, respectively, and a decrease in cholinergic neurotransmission at these sites in SIDS infants potentially reflect an impairment of arousal responses. With regard to sleeping/waking, the functional significance of a nAChR deficiency in the PoO and LC (marginally decreased) of the SIDS cases compared to controls is unknown. However, given a postulated role of the PoO in the generation of rapid eye movement (REM) sleep (64), we speculate that this lesion may interfere with REM–non-REM (NREM) sleep regulation and the generation of REM sleep in SIDS cases with associated dysfunction in breathing and/or heart rate. Similarly, the LC's involvement in sleep to wake transitions and arousal (65) further support a role for dysfunctional sleep regulation as part of the pathogenesis of SIDS. While the current finding of decreased binding in the PoO and LC in SIDS differs from previous studies that found no change at these sites, these pontine sites were previously identified as affected by maternal smoking (19), thus supporting their inherent vulnerability to alterations in cholinergic development and potentially function.

### Effects of Pre-natal Exposure on ^125^I-Epibatidine Binding in the Infant Brainstem

A major finding in this study is that pre-natal smoking is associated with an increase in ^125^I-epibatidine binding in SIDS and controls combined in 3 sites of the rostral medulla after controlling for PCA—the GC, RO, and PGCL. Additional analyses show that this significant effect is being driven mainly by post-KCOD controls, particularly in the RO and PGCL where smoking significantly increases ^125^I-epibatidine binding in the controls but not in SIDS. Our data of increased receptor binding in response to pre-natal smoking are consistent with the literature (see below) and suggests a compensatory upregulation of ^125^I-epibatidine-labeled nAChRs, possibly in response to a decrease in cholinergic activity at these sites (66, 67). Further studies on other cholinergic markers in this dataset are necessary to address the compensatory relationship between cholinergic markers and nAChRs in our dataset. It is of interest that the significant increase in ^125^I-epibatidine binding, seen predominately in controls, occurred in three of the five medullary nuclei that are significantly decreasing with age. This represents complex and dynamic neuroplastic changes associated with both development and exposure. The fact that the effect of exposure in the SIDS infants was not statistically significant at these sites suggests a lack of compensatory neuroplasticity within these sites in SIDS. Whether the normal mechanisms shown to be involved in upregulation of nAChRs [post-translational receptor assembly (68), trafficking (68), cell surface expression (68), degradation (68), and affinity alterations (69); post-transcriptional modification by microRNAs (70)] are deficient in SIDS is unknown; however, the potential consequences of incomplete compensation are noteworthy.

Given that acetylcholine modulates serotonergic activity in regulation of cardiorespiratory and homeostatic functions (71–74), an imbalance of acetylcholine transmission (via abnormal nicotinic and/or muscarinic receptors) in SIDS infants likely puts them at risk for sudden death in response to homeostatic challenges. As noted, embedded within the GC, PGCL, and RO are 5-HT neurons. Our data showing an effect of pre-natal smoking in these nuclei support literature reporting an effect of pre-natal nicotine on 5-HT neuron neurotransmission in general (67, 75–77) and directly on medullary 5-HT neurons (78, 79). Numerous experimental and human studies [reviewed in Vivekanandarajah et al. (36)] have shown that maternal cigarette smoke/nicotine exposure adversely affects ventilation (80), breathing drive (81), respiration rate (82–84), ventilatory drive (85, 86), respiratory rhythm pattern generation (87), and arousal responses to adverse stimuli such as hypoxia and hypercapnia (88–91)—responses mediated in part by medullary 5-HT neurons within the RO, GC, and PGCL. It is important to note that maternal smoking exposes a fetus to more than just nicotine, including toxins that have been shown to have unique neurodevelopmental effects and/or combine with nicotine to exacerbate nicotine's effect (66, 67). Given this, our findings are likely not attributed to nicotine alone.

Our data on the amount of pre-natal smoke exposure positively associated with post-natal nicotinic receptor binding are in agreement with the experimental animal models studying the brainstem. Animal models of chronic pre-natal exposure have generally shown increased nAChR expression in the infant brainstem (92–96). Maternal nicotine exposure during pregnancy have resulted in increased nAChR binding in mouse (97) and rhesus monkey (93), increased nicotinic receptor mRNA in the rat (95), and increased nAChR protein expression in the mouse (98) at various brainstem sites. Slotkin et al. also reported an overall increase in ^125^I α-bungarotoxin binding in the brainstem that emerged in the early post-natal period (99). Studies of human infant pre-natal exposures demonstrate conflicting results. Our laboratory previously reported an increase in ^3^H-nicotine binding in mesopontine nuclei related to cardiorespiration (nucleus parabrachialis) and arousal (LC and PoO) (19) and a decrease in binding in the LC nucleus, periaqueductal gray, and the raphe dorsalis, part of the ascending 5-HT arousal system (21). Other groups have reported decreased α7, β2 nAChR protein expression in the arcuate nucleus, XII, and NTS (22), decreased α4 nAChR fiber staining in the cribriform nucleus (24) in smoke-exposed infants, and increased α7 nAChR protein expression in major brainstem sites associated with pre-natal smoke exposure (100). The differences in the direction of change may be attributable to the composition of the control groups across the datasets with differing cohorts of infants who die from varying causes, the patterns of smoking, and/or the experimental measures, including the use of different ligands and antibodies. Irrespective of direction (up- or downregulation due to exposure), the specificity of change only in KCOD controls is consistent with our other studies showing alterations in controls but not in SIDS. Furthermore, these differences provide an example of the complexity of human autopsy studies where, without measurable cotinine levels, it is unclear whether the differences are due to acute changes in circulating nicotine close to the time of death or due to early or sustained developmental effects of nicotine exposure *in utero*.

Interestingly, there was no effect of the amount of maternal alcohol use on ^125^I-epibatidine binding in the brainstem. This differs from the Aberdeen Area Infant Mortality Study (AAIMS) showing a significant reduction in nicotinic receptor binding with an increase in the average number of drinks per month during pregnancy (21). It also differs from experimental studies, both *in vivo* (101–103) and *in vitro* (104), that have shown changes in nicotinic receptor expression following alcohol exposure. It is possible that our continuous measure of drinks per pregnancy is not discerning enough to detect small effects or effects that are trimester specific. It is also possible that the size of the cohort does not have the statistical power to detect effects of alcohol on ^125^I-epibatidine. With these caveats, however, our data do support that, in this cohort, pre-natal smoke exposure plays a greater role in altering nicotinic receptor binding than pre-natal alcohol exposure. Although the results of the present study show that amount of pre-natal alcohol exposure does not alter brainstem nicotinic receptors in infants, further studies are warranted to investigate the effect on other neurotransmitter systems, such as the serotonergic system.

#### Other Factors of Consideration

Many of the cases (KCOD and SIDS) had some degree of infection, either peripheral or central. In SIDS, this is consistent with minor infection prior to death as a known risk factor (105). The role of nAChRs, specifically the α7 subtype, in neuroinflammation is becoming more appreciated with regard to their function on inflammatory cells (106) and the changes seen in α7 neuronal expression in response to some pathogens and pathogen-specific proteins (107). While we cannot rule out a possible effect of inflammation on our data, given that epibatidine binds the α4β2 nAChR subtype with a much greater affinity than the α7 nAChR subtype (108), an effect of inflammatory-associated influence on α7 nAChR is likely to be minimal. Post-natal exposure to cigarette smoke is another potential consideration (22) as is the effects of pre-natal exposure to cannabinoids (109) from marijuana and methamphetamine (110). Given the relatively small number of cases across SIDS and KCOD controls reportedly exposed postnatally to cigarette smoke (*n* = 5) and prenatally to marijuana and/or methamphetamine (*n* = 4), we did not assess their influence on our data. Larger datasets with quantitative measures of these exposures are necessary to separate out an independent or potentially synergistic influence of these exposures on nAChR binding. Finally, intermittent episodes of hypoxia either prenatally or postnatally have been proposed in the pathogenesis of SIDS (61). While we have no means to identify or quantitate hypoxic events in our cases, the potential influence of hypoxia on nAChR expression should be noted (111–113).

### Limitations of the Study

A major limitation of this study is the relatively small sample size. The validity of results is supported, however, by their general consistency with previously reported data in other cohorts and animal models. A second limitation of this study is the fact that all SIDS, nearly all post-KCOD controls (90%), and all pre-KCOD controls were exposed to pre-natal smoking to some degree, thus making it difficult to detect differences in binding between SIDS and post-KCOD controls controlling for exposure. Despite this, the continuous values measuring pre-natal exposure to smoking enabled us to determine the association between the amount of exposure and nicotinic receptor binding and the developmental trajectory of nicotinic receptor binding with age. A third limitation is that the autopsied control infants were not representative of living controls. This limitation is not specific to the Safe Passage Study but is inherent in all autopsy case/control studies. Another limitation of the study is that there was no experimental verification of the biological concentration of pre-natal exposure in the infant's system. The pre-natal exposures related to smoking and alcohol was based on the mother's self-report of her consumption without verification with biomarkers such as cotinine measurements for smoke exposure and ethanol metabolites for alcohol exposure in infant blood. Finally, the selected radioligand ^125^I-epibatidine binds with most, but not optimally, or with all nicotinic receptor subtypes. Additional analysis of and comparison between ^125^I-bungarotoxin and ^3^H-nicotine would allow for a more comprehensive analysis of nAChRs in the brainstem (114, 115).

## Conclusions

In summary, our data confirmed our hypothesis that nAChR binding is abnormal in SIDS infants compared to infants dying of known causes. Although significant difference was only detected in one nucleus, the PoO, this abnormality represents a deficit in an arousal system that likely places an infant at risk for SIDS. The fact that deficiencies were only found in one nucleus supports that other neurotransmitters, including 5-HT, may be more affected in SIDS in this database. Relevant to pre-natal exposures, our data support that SIDS infants are not properly responding to or compensating for an effect of pre-natal nicotine exposure by increasing nicotinic receptors. This deficiency, especially in medullary nuclei containing 5-HT neurons, could hinder a normal adaptive/neuroplastic mechanism in SIDS that extends into the post-natal period and decreases the effectiveness of homeostatic responses. Our data showing an effect of pre-natal smoking in medullary nuclei containing 5-HT neurons support the vulnerability of these sites, previously identified as abnormal in SIDS by serotonergic measures, to adverse developmental exposures. The vulnerability is likely heightened during the first year of life when post-natal developmental changes in 5-HT and acetylcholine systems converge. In the Safe Passage Study, we recently reported a synergistic effect of maternal smoking and drinking on SIDS risk—an effect greater than either exposure alone (33). The results reported here showing no effect of maternal drinking on nAChRs suggest that the neuropathological basis for this combined exposure likely involves other systems, including other neurotransmitter systems within the brain. Overall, our results contribute to the wealth of other data, human and animal, supporting the evidence for the adverse effects of pre-natal exposures on a developing fetus, effects that persist into post-natal life, including the period of risk for SIDS.

## Data Availability Statement

The datasets presented in this article are not readily available because the data is not all publicly available due to regulatory agreements with American Indian tribal nations. Requests for data, however, will be reviewed on a case by case basis. Requests to access the datasets should be directed to robin.haynes@childrens.harvard.edu.

## Ethics Statement

The studies involving human participants were reviewed and approved by Health Research Ethics Committee of Stellenbosch University. Other institutional review board approvals, including tribal review boards for reservation-based sites in the Northern Plains, were obtained for all PASS entities (clinical sites, and centers for data coordination, pathology, and physiology). The research was overseen by the Network's Steering Committee and an external Advisory and Safety Monitoring Board. Written informed consent to participate in this study was provided by the participants' legal guardian/next of kin.

## Author's Note

The authors of this manuscript from the Safe Passage Study would like to dedicate it to a beloved member of our group, Johan Dempers, who died of COVID-19 days before this manuscript was accepted for publication. Johan Dempers served on the front lines as the Head of Forensic Medicine at the Western Cape Forensic Pathology Service, Tygerberg, and the Faculty of Medicine and Health Sciences of Stellenbosch University in Cape Town, South Africa.

The Safe Passage Study could not have been completed without Johan's leadership and grasp of the impact of infant mortality on his community. He was motivated to pursue SIDS research by a love of children, having two beautiful children of his own. He was passionate about finding the cause of SIDS, especially among disenfranchised groups in South Africa. He was described by many as larger than life, with wide and varied interests, including reading Shakespeare, playing drums in a band (with his daughter as singer and bass guitar) and traveling the world with his beloved family: his wife Karen, daughter Mieneke, and son Daniël. He was proud of his Afrikaans language and heritage and his country, acknowledging its faults of the past to assure a better and more educated tomorrow. He was a wise and enthusiastic scholar and teacher of forensic medicine, a man who loved life and held it sacred in the most difficult settings, including the tragedy of infant death. He took on some of the most challenging, hardened and complex cases in the courtroom with an unshakeable sense of fairness and both academic knowledge and experience to guide the legal profession. He was never without a smile, a word of encouragement, a hearty laugh and the ability to relate to all. His kindness and philanthropy were constant and personal—assisting a friend or stranger just because he could meet a need. We were privileged to know him and to work with him. He became a friend to us all, without exception, and we will greatly miss his joyful and all-inclusive ways.

## Author Contributions

RH: had full access to all the data in the study and takes responsibility for the integrity of the data and the accuracy of the data analysis. HK, AE, WF, MMM, HO, and RH: concept and design. AV, RH, HK, MN, AE, RF, HT, JC, PJ, MM, KM, JRD, KB, KS, HO, JA, LB, EB, JAC, JDD, TB, WF, EG, CG, IH, MMM, BR, PS, MS, CW, DR, LN, DZ, and SW: acquisition, analysis, or interpretation of data. RH, AV, HK, and MN: drafting of the manuscript. RH, AV, HK, MN, AE, HO, WF, MMM, RF, and JRD: critical revision of the manuscript for important intellectual content. MN: statistical analysis. HK, AE, WF, HO, and RH: obtained funding. AV, RH, HK, AE, WF, MMM, HO, RF, HT, JC, PJ, MM, KM, JRD, JA, KB, and KS: administrative, technical, or material support. RH, HK, AE, WF, HO, JA, RF, and CG: supervision. All authors approved the final manuscript as submitted and agree to be accountable for all aspects of the work.

## Conflict of Interest

The authors declare that the research was conducted in the absence of any commercial or financial relationships that could be construed as a potential conflict of interest.
