# Supplementary material for: Nicotinic Receptors in the Brainstem Ascending Arousal System in SIDS With Analysis of Pre-natal Exposures to Maternal Smoking and Alcohol in High-Risk Populations of the Safe Passage Study
Source: Front Neurol. 2021 Mar 10;12:636668. doi: 10.3389/fneur.2021.636668 (PMC7988476; doi:10.3389/fneur.2021.636668)
Supplement: Supplementary file 1 [file Presentation_1.pdf]

Supplemental Figure. Anatomical relationship between medullary and pontine nuclei as they relate to serotonergic and cholinergic neuronal distribution. Nuclei containing serotonergic neurons are colored green, nuclei containing cholinergic neurons are colored red. Other nuclei shown with no color either contain different neurotransmitters (i.e, norepinephrine in the locus coeruleus [LC]) or a mixture of neurotransmitter types. Cholinergic neurons within the pons project diffusely (projections not shown) to medullary nuclei involved in cardiorespiratory control. The function of medullary and pontine nuclei in cardiorespiratory control, arousal, and sleep are described below to aid in evaluating the relevance of reported findings.

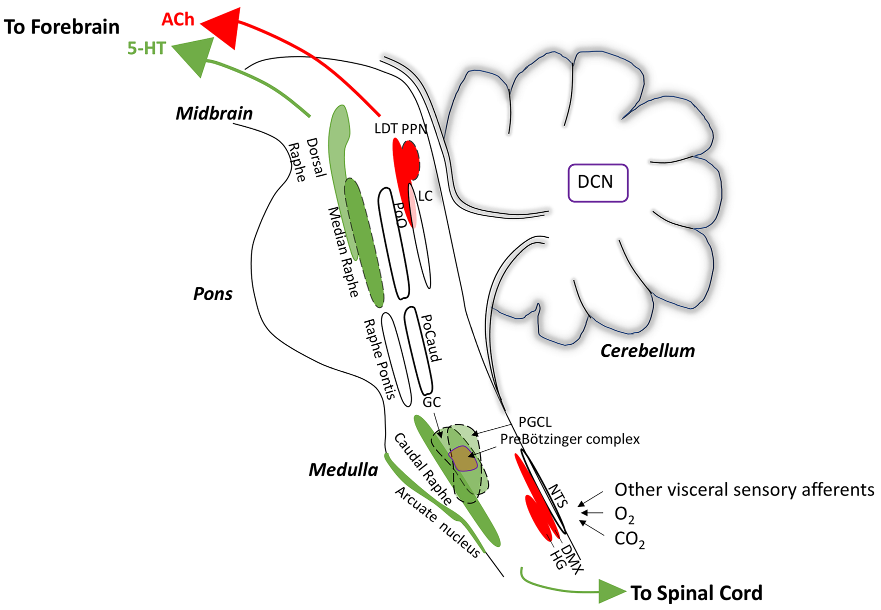

Supplemental Text.

Medullary cardiorespiratory control. Cardiorespiratory activity and arousal are controlled by a network of neurons located in the lower brainstem, i.e., medulla and pons (Spyer and Gourine, 2009). Concentrated in the medulla are subnetworks that are essential for survival and regulate breathing, blood pressure, heart rate, upper airway reflexes, gasping, vomiting, and respiratory skeletal muscle coordination in the airway, chest, and abdomen for synchronous breathing, in part via the ponto-olive-cerebellum subnetwork (Parsons et al., 2001). The basic rhythm of breathing is generated by subnetworks within the Pre-Bötzinger complex in the medulla which is postulated to be embedded in the ventrolateral reticular formation in the human, and to be putatively homologous to the human paragigantocellularis lateralis (PGCL) with the caveat that it contains scattered serotonergic neurons unlike rodents (Paxinos et al., 2012; Kinney and Haynes, 2019). The Pre-Bötzinger complex is modulated by pontine and other inputs from cell groups within the medulla, and then transmitted to pre-motor neurons in the spinal cord that relay the respiratory pattern to cranial and spinal motor neurons controlling respiratory muscles (Spyer and Gourine, 2009). Cardiovascular sympathetic and vagal (autonomic nervous system) activities have characteristic discharges that are patterned by respiratory activity (Spyer and Gourine, 2009). This patterning ensures ventilation-perfusion matching for optimal respiratory gas exchange in the lungs (Spyer and Gourine, 2009). Peripheral arterial and central respiratory chemoreceptors are vital for the maintenance of cardiorespiratory homeostasis; inputs from these receptors mediate adaptive changes in cardiorespiratory motor outputs in changing physiological and pathological

conditions (Spyer and Gourine, 2009). Of note, suprapontine structures play a role in patterning autonomic outflows according to different behaviors (Spyer and Gourine, 2009). The level of arterial pCO2 is the primary factor in determining respiratory drive (Spyer and Gourine, 2009) and the principal sensors are located either at or close to the ventral surface of the medulla, e.g., retrotrapezoid nucleus. The human arcuate nucleus is postulated to be homologous to the respiratory chemosensitive fields at the ventral medullary surface in the cat (Zec et al., 1997). The medullary serotonergic subnetwork is comprised of serotonin (5-HT) cell bodies in the medulla and their projection sites to nuclei that modulate cardiorespiratory control (Kinney et al., 2009). Nicholls and Paton (Nicholls and Paton, 2009) maintain that neural networks regulating respiratory, cardiac activity, and blood pressure are so "interwoven" within the medulla that the medulla best be considered as a "single system".

Arousal. We measured arousal-related nuclei in the pons and medulla. They were each part of the ascending arousal network (ANN) that mediates the switch from sleep to waking and sustains the waking state via connections to cerebral networks (Edlow et al., 2012). These interacting networks mediate awareness and arousal which are the neural basis of consciousness. There are several neurotransmitter-specific systems whose fibers project differentially and rostrally from the brainstem, hypothalamus, or basal forebrain to mediate arousal by interconnecting with awareness networks in the cerebral cortex. These transmitter-specific systems are the serotonergic neurons arising from the raphe and extra-raphe in the brainstem; cholinergic neurons arising from the mesopontine reticular formation [pedunculopontine nucleus (PPN) and laterodorsal tegmental nuclei (LDT)] and projecting mainly to the basal forebrain and not the cerebral cortex; noradrenergic neurons arising from the LC, as well as other systems recently reviewed (Scammell et al., 2017). These systems mediate different aspects of multifaceted arousal, yet there also appears to be some redundancy to ensure protection of this vital function. The pathways of the AAN are interconnected to the cardiorespiratory nuclei in the brainstem to coordinate homeostatic functions, like breathing and heart rate with the level of state (e.g., waking, sleep, fright, or flight) (Edlow et al., 2012).

REM sleep and NREM sleep. There are several models of the regulation of rapid eye movement (REM) and non-REM (NREM) sleep, and theories are rapidly changing with advancing discoveries (Scammell et al., 2017). Consciousness fades and sleep moves into the deep stages of NREM sleep; sensory gating blocks all but the strongest and most salient stimuli (Scammell et al., 2017). During REM sleep, dreams occur, and are accompanied by rapid eye movements and fluctuations in breathing and heart rate. Mutual inhibition between these specific pathways give rise to REM and NREM states, and dysfunction in these circuits can lead to sleep derangements (Scammell et al., 2017). The cholinergic system within the pontis oralis (PoO) of the pontine rostral reticular formation is critically involved in the generation of REM sleep (Xi et al., 2004; Rodrigo-Angulo et al., 2005). Cholinergic agonists, when injected into the PoO are capable of inducing a REM-like state with all of its attendant physiologic patterns of activity (Baghdoyan et al., 1984; Yamuy et al., 1993; Xi et al., 2004). The excitatory cholinergic activation of PoO neurons that are involved in the generation of REM sleep is gated by a pontine gamma aminobutyric acid (GABA)ergic inhibitory system that exerts its effects postsynaptically by inhibiting PoO neurons, resulting in the suppression of REM sleep and the generation of wakefulness (Xi et al., 2004). In the absence of the activation of this GABAergic gating mechanism, REM sleep occurs.

Bibliography

Baghdoyan, H.A., Rodrigo-Angulo, M.L., McCarley, R.W., and Hobson, J.A. (1984). Site-specific enhancement and suppression of desynchronized sleep signs following cholinergic stimulation of three brainstem regions. *Brain Res* 306(1-2)**,** 39-52. doi: 10.1016/0006-8993(84)90354-8.

Edlow, B.L., Takahashi, E., Wu, O., Benner, T., Dai, G., Bu, L., et al. (2012). Neuroanatomic connectivity of the human ascending arousal system critical to consciousness and its disorders. *J Neuropathol Exp Neurol* 71(6)**,** 531-546. doi: 10.1097/NEN.0b013e3182588293.

Kinney, H.C., and Haynes, R.L. (2019). The Serotonin Brainstem Hypothesis for the Sudden Infant Death Syndrome. *J Neuropathol Exp Neurol* 78(9)**,** 765-779. doi: 10.1093/jnen/nlz062.

Kinney, H.C., Richerson, G.B., Dymecki, S.M., Darnall, R.A., and Nattie, E.E. (2009). The brainstem and serotonin in the sudden infant death syndrome. *Annu Rev Pathol* 4**,** 517-550. doi: 10.1146/annurev.pathol.4.110807.092322.

Nicholls, J.G., and Paton, J.F. (2009). Brainstem: neural networks vital for life. *Philos Trans R Soc Lond B Biol Sci* 364(1529)**,** 2447-2451. doi: 10.1098/rstb.2009.0064.

Parsons, L.M., Egan, G., Liotti, M., Brannan, S., Denton, D., Shade, R., et al. (2001). Neuroimaging evidence implicating cerebellum in the experience of hypercapnia and hunger for air. *Proc Natl Acad Sci U S A* 98(4)**,** 2041-2046. doi: 10.1073/pnas.98.4.2041.

Paxinos, G., Haung, X., Sengul, G., and Watson, C. (2012). "Organization of Brainstem Nuclei," in *The Human Nervous System,* eds. J. Mai & G. Paxinos. Third Edition ed (London, UK: Academic Press), 260-327.

Rodrigo-Angulo, M.L., Rodriguez-Veiga, E., and Reinoso-Suarez, F. (2005). A quantitative study of the brainstem cholinergic projections to the ventral part of the oral pontine reticular nucleus (REM sleep induction site) in the cat. *Exp Brain Res* 160(3)**,** 334-343. doi: 10.1007/s00221-004-2015-x.

Scammell, T.E., Arrigoni, E., and Lipton, J.O. (2017). Neural Circuitry of Wakefulness and Sleep. *Neuron* 93(4)**,** 747-765. doi: 10.1016/j.neuron.2017.01.014.

Spyer, K.M., and Gourine, A.V. (2009). Chemosensory pathways in the brainstem controlling cardiorespiratory activity. *Philos Trans R Soc Lond B Biol Sci* 364(1529)**,** 2603-2610. doi: 10.1098/rstb.2009.0082.

Xi, M.C., Morales, F.R., and Chase, M.H. (2004). Interactions between GABAergic and cholinergic processes in the nucleus pontis oralis: neuronal mechanisms controlling active (rapid eye movement) sleep and wakefulness. *J Neurosci* 24(47)**,** 10670-10678. doi: 10.1523/JNEUROSCI.1987-04.2004.

Yamuy, J., Mancillas, J.R., Morales, F.R., and Chase, M.H. (1993). C-fos expression in the pons and medulla of the cat during carbachol-induced active sleep. *J Neurosci* 13(6)**,** 2703-2718.

Zec, N., Filiano, J.J., and Kinney, H.C. (1997). Anatomic relationships of the human arcuate nucleus of the medulla: a DiI-labeling study. *J Neuropathol Exp Neurol* 56(5)**,** 509-522.
